# Hierarchical Thin Film Architectures for Enhanced Sensor Performance: Liquid Crystal-Mediated Electrochemical Synthesis of Nanostructured Imprinted Polymer Films for the Selective Recognition of Bupivacaine

**DOI:** 10.3390/bios4020090

**Published:** 2014-04-08

**Authors:** Subramanian Suriyanarayanan, Hazrat Nawaz, Natacha Ndizeye, Ian A. Nicholls

**Affiliations:** 1 Bioorganic and Biophysical Chemistry Laboratory, Linnæus University Centre for Biomaterials Chemistry and Department of Chemistry and Biomedical Sciences, Linnæus University, SE-391 82 Kalmar, Sweden; E-Mails: esusu@lnu.se (S.S.), nawazhazrat@gmail.com (H.N.); natacha.ndizeye@lnu.se (N.N.); 2 Department of Chemistry—BMC, Uppsala University, Box 576, SE-751 23 Uppsala, Sweden

**Keywords:** bupivacaine, electropolymerization, liquid crystal, molecularly imprinted polymer, nanostructured polymer films, piezoelectric sensor, quartz crystal microbalance

## Abstract

Nanostructured bupivacaine-selective molecularly imprinted 3-aminophenylboronic acid-*p*-phenylenediamine co-polymer (MIP) films have been prepared on gold-coated quartz (Au/quartz) resonators by electrochemical synthesis under cyclic voltammetric conditions in a liquid crystalline (LC) medium (triton X-100/water). Films prepared in water and in the absence of template were used for control studies. Infrared spectroscopic studies demonstrated comparable chemical compositions for LC and control polymer films. SEM studies revealed that the topologies of the molecularly imprinted polymer films prepared in the LC medium (LC-MIP) exhibit discernible 40 nm thick nano-fiber structures, quite unlike the polymers prepared in the absence of the LC-phase. The sensitivity of the LC-MIP in a quartz crystal microbalance (QCM) sensor platform was 67.6 ± 4.9 Hz/mM under flow injection analysis (FIA) conditions, which was ≈250% higher than for the sensor prepared using the aqueous medium. Detection was possible at 100 nM (30 ng/mL), and discrimination of bupivacaine from closely related structural analogs was readily achieved as reflected in the corresponding stability constants of the MIP-analyte complexes. The facile fabrication and significant enhancement in sensor sensitivity together highlight the potential of this LC-based imprinting strategy for fabrication of polymeric materials with hierarchical architectures, in particular for use in surface-dependent application areas, e.g., biomaterials or sensing.

## 1. Introduction

In surface-based sensing technologies such as quartz crystal microbalance (QCM), surface plasmon resonance (SPR), total internal reflectance fluorescence spectroscopy (TIRF) and electrochemical sensing, the proximity of the analyte to the transducer surface is critical for the sensor response [1,2,3,4,5,6]. Sensor surfaces based upon thin polymer film coatings have become of increasing interest in this regard due to their stability, breadth of polymer functionalities available and the possibility of regulating film thickness, e.g., when using electrochemical [7,8,9] or INIFERTER-based synthesis strategies. 

Molecularly imprinted polymer (MIP) [10,11,12,13,14] films have attracted significant interest for use in such sensor platforms due to the possibility of tailoring the ligand recognition characteristics of the material through the templating process, together with the advantages described above [15,16,17,18]. Despite the success achieved in sensing using MIP-based thin films, the need for greater sensitivity remains, in particular for applications where the analyte is present in very low concentrations, e.g., biomarkers, some toxins. Recently, efforts have been directed towards manipulating the morphologies of MIP-thin films in order to optimize both the availability of binding sites and analyte diffusion to these sites [19,20]. A number of strategies are evolving for creating hierarchical architectures in these materials through a combination of nanostructuring and molecular imprinting, to afford predetermined polymer structural features in both the Ångström–nanometer and nanometer-micrometer scales [21,22,23,24]. In the studies reported to date, *top-down* processes such as lithography and the use of sacrificial structures have been used to steer morphologies. *Bottom-up* approaches have been limited to the choice of polymer or solvent (often referred to as the porogen), and the regulation of thickness when using INIFERTER or electrochemical synthesis strategies. 

We are currently exploring new strategies for directing the morphologies of imprinted polymeric materials [24], ideally such strategies should be economically viable and offer the potential for scale-up. In this study we have explored a *bottom-up* strategy where liquid crystalline (LC) structures are used to impart nano-scale structuring in molecularly imprinted polymer films and we have examined the impact of the resulting morphological features on the performance of films imprinted with the local anesthetic bupivacaine when used in a QCM platform.

## 2. Experimental Section

### 2.1. Chemicals

**Scheme 1:**
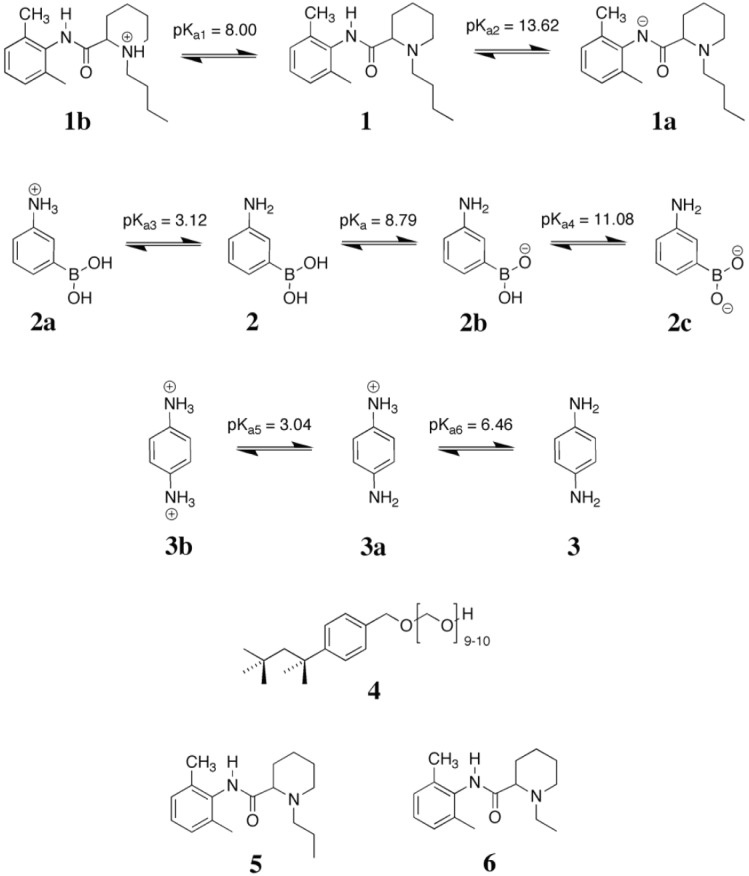
Protonation/deprotonation dissociation equilibria of bupivacaine (**1**) and monomers employed: *3-*aminophenylboronic acid (**2**) and *p*-phenylenediamine (**3**). Structural formula of Triton X-100 used for the preparation of liquid crystalline medium: polyethylene glycol *p*-(1,1,3,3-tetramethylbutyl)-phenyl ether (**4**). Template analogues used to evaluate sensor performance: mepivacaine (**5**) and ropivacaine (**6**).

Bupivacaine hydrochloride (**1**), 1,3-aminophenylboronic acid (**2**), *p*-phenylenediamine (**3**), Triton X-100 (**4**), mepivacaine hydrochloride (**5**), ropivacaine hydrochloride (**6**) (see Scheme 1), sodium sulfate (Na_2_SO_4_), sodium hydroxide, sodium chloride, sodium dihydrogen phosphate (NaH_2_PO_4_), disodium hydrogen phosphate (Na_2_HPO_4_) and sulfuric acid were procured from Sigma-Aldrich (Steinheim, Germany). Hydrogen peroxide (30%) was obtained from Fluka (Buchs, Switzerland). A Milli-Q gradient water filtration system (Millipore, MA, USA) was used to purify distilled water to ultrapure grade with resistance values of ≤18.2 MΩ Ultrapure water-Milli Q water was used for solution preparations and for the rinsing of substrates. 

### 2.2. Instrumentation and Protocols

#### 2.2.1. Electrochemistry and Quartz Crystal Microbalance

Electrochemical syntheses were performed with cyclicvoltammetry (CV) (see Section 2.3 for sensor fabrication) using a Reference 600 potentiostat/galvanostat (Gamry instruments, Warminster, PA, USA). The Gamry framework software provided by the manufacturer was used to control the instrument. Platinum wire and Ag|AgCl electrodes supplied by Gamry instruments were used as counter and reference electrodes, respectively.

Piezoelectric microgravimetric measurements were performed using a quartz crystal microbalance (QCM) system (Attana A100, Attana AB, Stockholm, Sweden). The QCM was configured for flow injection analysis (FIA) and was controlled by the Attester software supplied by the maker. 10 MHz AT-cut quartz resonators (Attana AB, Stockholm, Sweden) of 8 mm diameter, sputtered with 45 mm diameter and 140 nm thick gold on both the sides (adhered with a 10 nm Ti or Cr underlying layer), was used both as the working electrode and substrate sensor evaluation. Substrates were cleaned by immersion in piranha solution (1:3, *v*/*v*; H_2_O_2_:H_2_SO_4_) for 1 min (*caution: the piranha solution reacts violently with organic compounds and contact with the skin or eyes is dangerous*). The substrate was then rinsed extensively with ultrapure water, dried under a stream of N_2_ gas and stored under vacuum until use.

#### 2.2.2. Scanning Electron Microscopy (SEM)

Electron micrographs of electrodeposited polymer films were imaged using a Leo 1550 Gemini instrument furnished with a field emission electron gun in the high vacuum mode. The substrates were placed on a black carbon tape attached to alumina stubs and coated with a thin layer of platinum using a platinum sputtering unit (LEICA EM SCD 500) before being inserted into the SEM instrument. The pressure of the measurement chamber was maintained at 2 × 10^−5^ mbar. A 3 kV potential was applied to the electron gun to generate the electron beam used to scan the sample surface.

#### 2.2.3. UV-Visible Spectroscopy

UV-visible spectra were measured in the range 350–200 nm at 0.1 nm resolution by means of UV-1800 spectrophotometer of Shimadzu Corporation (Tokyo, Japan).

#### 2.2.4. IR Spectroscopy

RAIR spectra of the polymer film coated Au/quartz surface were recorded in a Bruker Hyperion 3000 IR microscope coupled to a Tensor 27 IR spectrometer and computerized sample stage. Infrared beam was double reflected from the surface with angles 52° and 83° to the surface normal using a grazing angle objective. The spectra were derived from 1000 interferograms collected by a single element mercury-cadmium-telluride (MCT) detector with a 4 cm^−1^ resolution. The sample chamber was maintained under an inert atmosphere throughout the measurement by purging with nitrogen gas at positive pressure. A three-term Blackmann–Harris apodization function was applied to the interferograms, prior to the Fourier transformation. An unmodified Au/quartz resonator was used to measure the background spectra. 

#### 2.2.5. Flow Injection Analysis

The sensing capabilities of the polymer film coated Au/quartz resonators were studied under flow injection analysis (FIA) conditions using a QCM (see Section 2.2.1). The substrates were mounted in the flow cell holders provided by the manufacturers. Phosphate buffer (10 mM) containing 150 mM NaCl at pH 8.5 was used as the carrier buffer. A dual piston peristaltic pump, in-built within the QCM instrument was used to pump the carrier buffer solution over the polymer film coated substrates at the desired flow rates. The buffer solution was allowed to equilibrate with polymer film at this condition to have minimum change (±0.5 Hz) in the resonant frequency for over 400 s. An aliquot (75 µL) of the test solution, diluted with carrier buffer was injected in the flow cell using a 6-point injection valve present in the instrument.

### 2.3. Sensor Fabrication and Characterization

MIP recognition films were prepared by electropolymerization on gold-coated quartz (Au/quartz) transducers. Initially, polymerization reaction mixtures were prepared by mixing **1** (bupivacaine, template), **2** and **3** (cross-linking functional monomers) in the ratio 1:5:25 and dissolved either in ultrapure water containing 0.2 M of Na_2_SO_4_ (supporting electrolyte) or LC medium comprising 42% of Triton X-100 in water (*v*/*v*). The solutions were allowed to equilibrate for 5 min prior to use. 

Piranha-cleaned Au/quartz resonators were mounted in the QCM chip holders (Attana AB, Stockholm, Sweden). A well-shaped groove was carved into the Perspex cover of the chip holder to provide a volume of 5 mm diameter and 2.5 mm depth above the gold surface. Next, the contact leads for the Au/quartz substrate, provided in the QCM chip holder was electrically connected to the working electrode terminal of the Gamry potentiostat. A drop of the pre-polymerization solution was then placed over the Au/quartz substrate. The counter and reference electrodes were carefully dipped into the solution close to the gold surface though without making contact with the Au surface, or between the electrodes. 

MIP recognition film was deposited by scanning the potential from −0.5 to 1.45 V at 50 mV/s. The growth of the polymer was governed by the number of cycles. Afterwards, the polymer film was rinsed in ultrapure water to remove the physisorbed species. The bupivacaine template was repeatedly extracted from the MIP film with aqueous NaOH (5 mM, 1 mL, 5 times) for 1 h. UV-visible spectra of the washing solutions were used to examine the efficiency of template removed from the MIP film. A reference polymer film was prepared by an identical procedure to that described above, though in the absence of the template bupivacaine. 

## 3. Results and Discussion

In surface-based sensing techniques, such as QCM, SPR and TIRF, the proximity of the analyte binding or recognition event to the transducer surface is critical for the sensor response. Thin polymer film coatings of molecularly imprinted materials have become of increasing interest in this area due to their stability, possibilities of regulating their thickness during fabrication and the broad range of structures that are amenable for use as a template. A number of reports have been made where sacrificial structures, either organic or inorganic, are used to generate complementary morphologies in polymer matrices [25,26,27]. The often rigorous treatment necessary for subsequent removal of these sacrificial structures, e.g., HF, suggested to us the need for alternative approaches, ideally requiring mild removal protocols. We perceived that colloidal structures might provide a stable enough framework to impart complementary structures in a cross-linked polymer, while at the same time being easily dismantled simply by elution under suitable conditions. It has previously been demonstrated that mixtures of triton X-100 and water can form hexagonally ordered cylindrical micelles in lyotrophic liquid crystals [28], as demonstrated by X-ray diffraction studies [7]. It was this well studied system that we proposed to use as a basis for the present study. 

### 3.1. Electrochemical Preparation of MIP Films

**Figure 1:**
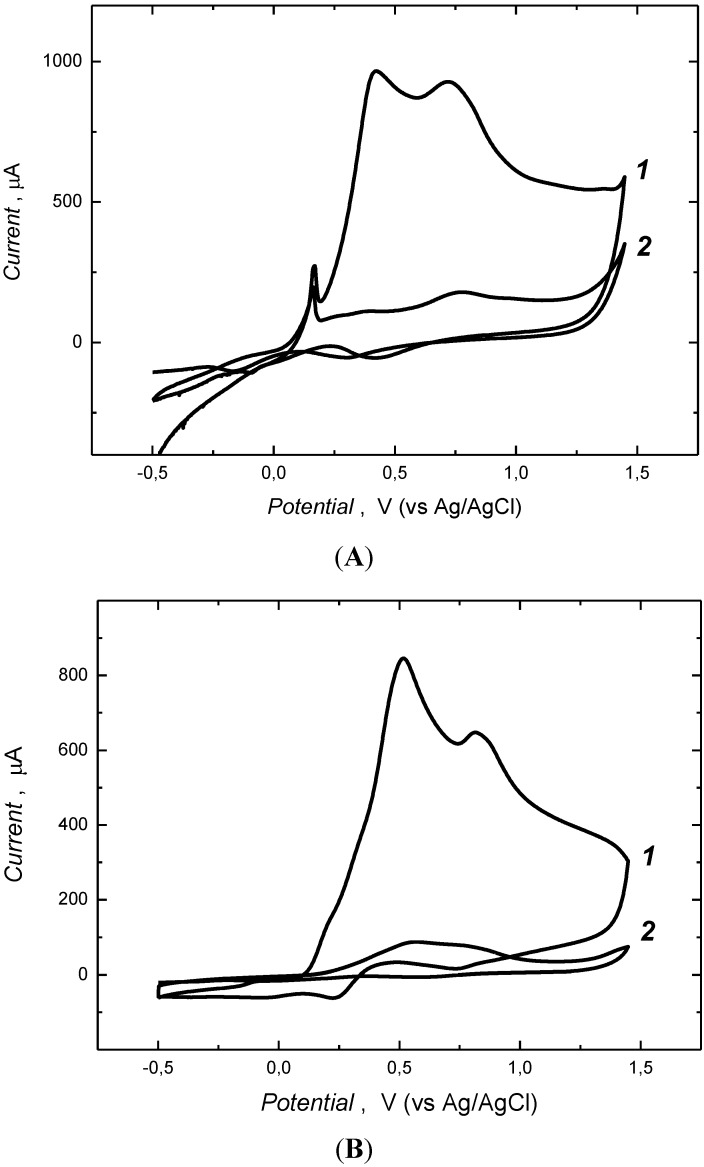
Cyclic voltammograms for the electrochemical co-polymerization of 3-APBA (**2**, 5 mM) and *p*-PD (**3**, 25 mM), in the presence (**A**) and absence (**B**) of 1 mM bupivacaine (**1**), in Triton X 100-water lytotropic liquid crystalline medium, containing 0.2 M Na_2_SO_4_ at pH 8.5, on the gold coated quartz electrode. Potential scan rate was 0.05 V/s. Curves *1* and *2* denote the first and fifth cycle of the cyclic voltammogram.

The liquid crystalline medium was prepared following established procedures [6] by heating triton X-100 (42%, v/v) in water to 50 °C under constant stirring to afford an isotropic phase. The mixture is then allowed to cool slowly to room temperature to form lyotrophic liquid crystalline phase. Polymerization reaction components; template (**1**) and the cross-linking functional monomers 1,3-aminophenylboronic acid (**2**, 3-APBA) and *p*-phenylenediamine (**3**, *p*-PD), were then mixed before being added to the LC medium. This particular polymer system was selected on the basis of results of another study that shall be communicated in elsewhere. Polymerization was performed CV. Figure 1A shows the CV curve for the copolymerization of **2** and **3** in the presence of the template (**1**) using Na_2_SO_4_ as supporting electrolyte on the Au/quartz electrode. The potential of the working Au/quartz electrode was ramped from −0.5 to 1.5 V at a 50 mV/s scan rate. In the anodic scan of the first cycle (Curve *1*, in Figure 1A), broad peaks around 0.5 V and 0.75 V can be attributed for the oxidation of 3-APBA and *p*-PD monomers to form radical cations [29,30,31,32]. Combined oxidation of bipolaron state of these radical cations is observed as a shoulder at 1.3 V, corresponding to the copolymerization [33,34]. The build up of the polymer film on the electrode surface is seen as a drastic decrease in the peak currents, for the oxidation of radical cations (Curve *2* in Figure 1A), owing to the hindered diffusion of the monomers to the electrode surface through. Growth of the pair of redox peaks at 0.74 V and 0.3 V, corresponding to the reduction and oxidation of the co-polymer film, respectively, further support this conclusion. A uniformly reddish brown gold electrode coated surface is obtained. For comparison, a reference polymer film (REF) was prepared identically, though in the absence of template. The similarities of the CV curve profiles from the preparation of the REF and MIP films discounts the possible that oxidation of bupivacaine takes place under the polymerization. 

**Figure A1:**
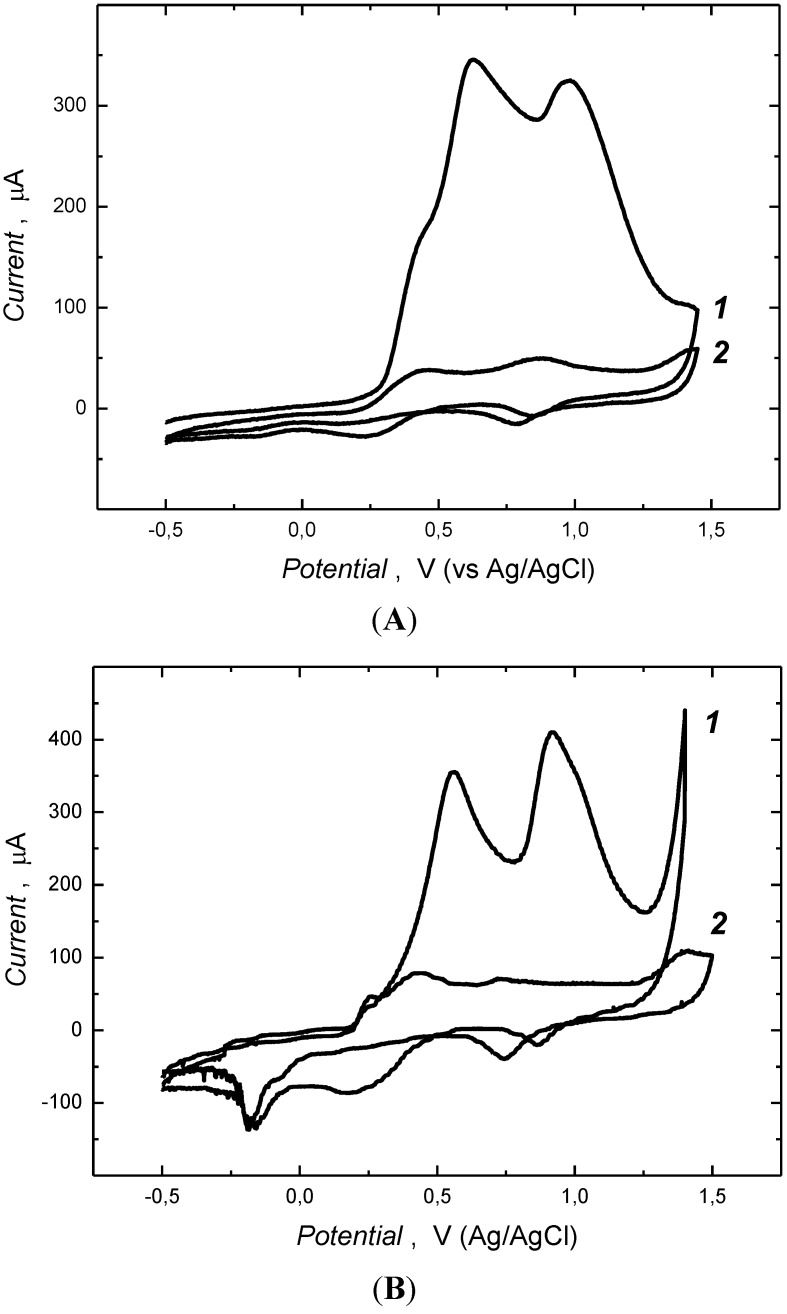
Cyclic voltammograms for the electrochemical co-polymerization of 5 mM of (**2**) and 25 mM of (**3**) in the presence (**A**) and absence (**B**) of 1 mM bupivacaine, in 0.2 M Na_2_SO_4_ aqueous solution at pH 8.0 on the gold coated quartz electrode. Potential scan rate was 0.05 V/s. Curves *1* and *2* denotes the first and fifth cycle of the cyclic voltammogram.

The CV curves for the electrodeposition of MIP and REF polymer films from water (Figure A1 in Appendix) shows similar features for the oxidation of cross-linking functional monomers and copolymerization, except the medium dependent shift in potentials and high current values. In the presence of the Triton X-100 the rate of polymerization is considerably enhanced due to various factors [35]. The polar head group (PEO chains) of the Triton X-100 may contribute by stabilizing the radical cations formation due to their attraction towards the oxygen atom of the PEO chains [36]. Apparently, this will decrease the monomer oxidation potential accelerating the polymerization. Further it has been argued that the PEO chains of the Triton X-100 adsorb on the electrode surface thus changing the electrode/electrolyte interface character to enable the facile diffusion of monomers towards to electrode surface [37]. This benefits the production of more cationic radicals and electropolymerization. Furthermore, triton X-100 improves the solubility of the monomers helping to afford well-defined growth of the polymer [36,38]. Here again, possible oxidation of template was ruled out as the CV curves profiles for MIP and REF polymers were essentially identical.

**Figure A2:**
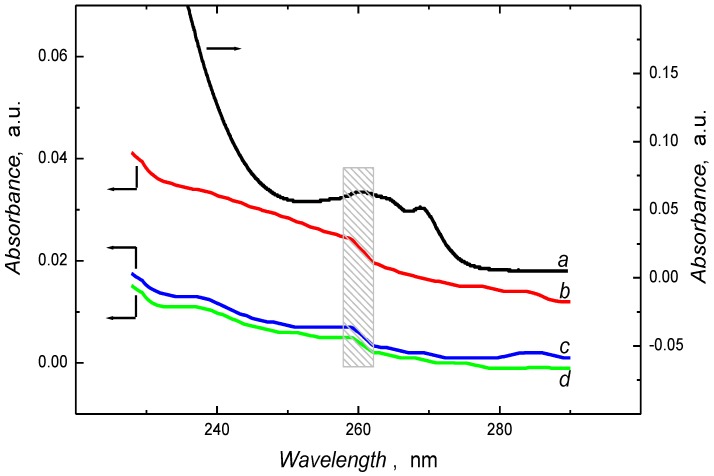
UV-vis spectra of 5 mM NaOH solution containing (*a*) 0.5 µM bupivacaine, (*b*) first, (*c*) second and (*d*) fourth extract of the bupivacaine templated MIP film. Bupivacaine was extracted from MIP film by washing with 5 mM NaOH.

The polymer films were washed with copious volumes of water to remove physisorbed materials, then extracted with a series of 5 mM NaOH(aq) solutions to remove the template. The efficiency of the template extraction process was monitored by UV-visible spectroscopy. The UV-vis spectra of the extract (Curves *b* and *c* in Figure A2) were recorded after rinsing the film for 1 h. The bands at 262 nm and 270.5 nm can be ascribed to bupivacaine (Curve *a* in Figure A2) [39,40]. After four consecutive extractions, the intensities of these bands diminish towards the baseline showing the practical completeness of template extraction process (Curve *d* in Figure A2). 

Topological and structural features of the polymer films were studied by microscopic (SEM) and spectroscopic (RAIRS) methods. Specific recognition of the polymer films towards bupivacaine and desired analytes were evaluated with QCM measurements.

### 3.2. Characterization of Polymer Films

**Figure 2:**
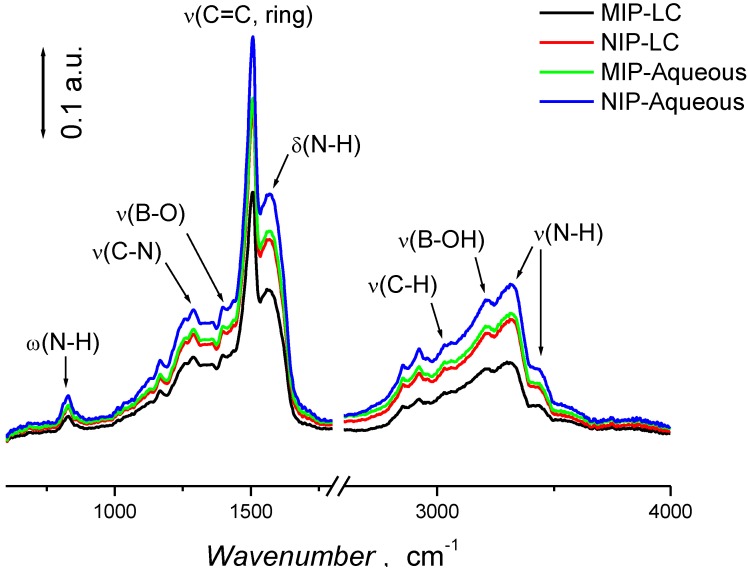
RAIR spectra of the bupivacaine MIP and REF films.

The chemical functionalities of the films were studied by refractive angle infrared (RAIR) spectroscopy. Figure 2 shows the RAIR spectra for MIP and REF films prepared in aqueous and LC medium. Discernible bands at 829, 1288, 1396, 1508, 1567, 3027, 3208, 3314, and 3442 cm^−1^ depict the ring bending, ν(C–N), ν(B–O), ν(C=C), δ(N–H), ν(C–H), ν(B–OH) and ν(N–H) IR-active vibrational modes present in the backbone of the copolymer [41,42,43,44]. In addition, the band at 1396 and broad bands around 3350 cm^−1^ corresponds to the stretching vibrations of –B–O [45] in 3-APBA and –NH_2_ moieties in *p*-PD, respectively, validate the copolymerization. The spectral features of the polymer films prepared in both media were very similar indicating that the polymerization reactions had incorporated both the monomers into the copolymer films. Interestingly, the lack of a significant difference between the spectra of the MIP and REF films indicates that the template does not affect the polymerization process and the copolymer composition. Furthermore, the absence of vibrational bands for the amide carbonyl (C(=O)NH) and ether (C–O–C) functionalities support the conclusion that the bupivacaine template and Triton X-100 were both efficiently removed from the polymer films.

**Figure 3:**
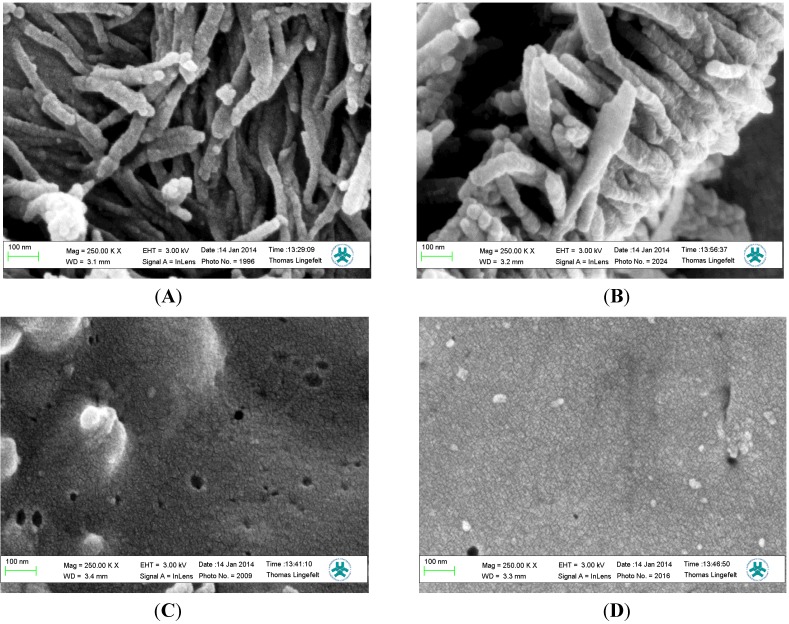
Surface topography mapped using scanning electron microscope (SEM) for the MIP (**A** and **B**) and REF film (**C** and **D**) coated on Au/quartz prepared in LC or aqueous media, respectively.

**Figure A3:**
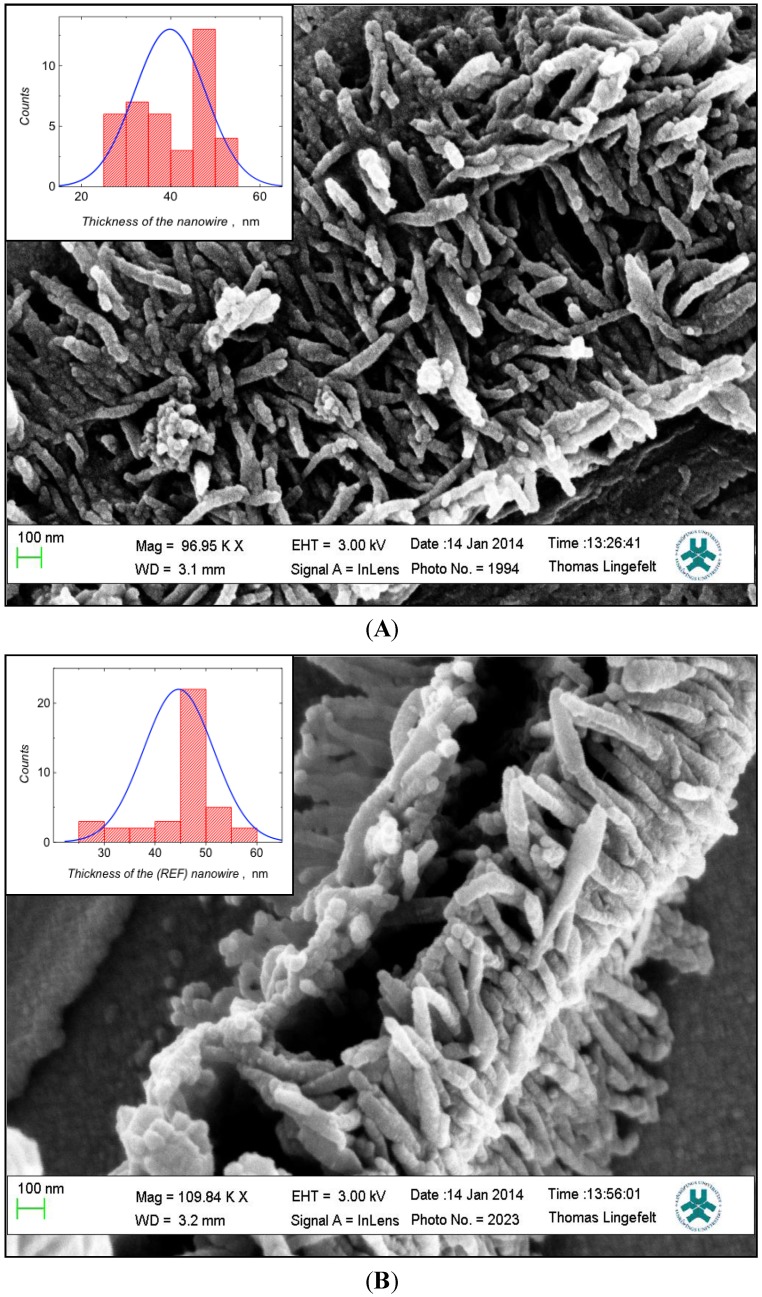
Scanning electron microscope (SEM) studies of the (**A**) MIP and (**B**) REF film coated Au/quartz resonators prepared in either LC or aqueous medium, respectively. Inset in (**A** and **B**) refers to the histogram of the thickness of the nanowires calculated with 40 entities from the SEM picture.

The topologies of the MIP and REF films were examined by SEM (Figure 3). Both the MIP and REF films prepared in the LC medium present unique morphological features with organized, densely packed fibrous nanowires or brush-like structures grown uniformly under electrochemical conditions. Figure A3 demonstrates the long-range uniformity of the nanowires with thickness ranging from 39.9 ± 7.8 nm and 44.6 ± 6.7 nm for MIP and REF films, respectively. The films prepared in aqueous media were uniform, and devoid of noticeable morphological features. The extreme increase in the surface areas of the polymer films as a result of using the LC medium is dramatic (Figure 3). The greater surface area afforded by fiber structures arising from the synthesis of the polymer films in the LC medium was anticipated to increase the number of accessible ligand binding cavities in close proximity to the sensor surface. 

### 3.3. Piezoelectric Microgravimetric (QCM) Determination of Bupivacaine

The influence of the polymer film hierarchical architecture, Ångström–nm sized cavities arising from the template and LC-phase induced morphologies at the nm–µm scale, on the polymer’s capacity to bind bupivacaine (**1**) was examined by QCM under FIA conditions. Studies were performed at pH 8.5 using a 0.01 M phosphate buffer containing 0.15 M NaCl as carrier solution. This pH was selected to limit the presence of charged functionalities present on polymer and analyte so as to reduce the impact of non-specific binding. Above this pH 3-APBA will be deprotonated (pK_a4_, Scheme 1) and below pH 8.0 bupivacaine is protonated (pK_a1_, Scheme 1) [46,47].

**Figure 4:**
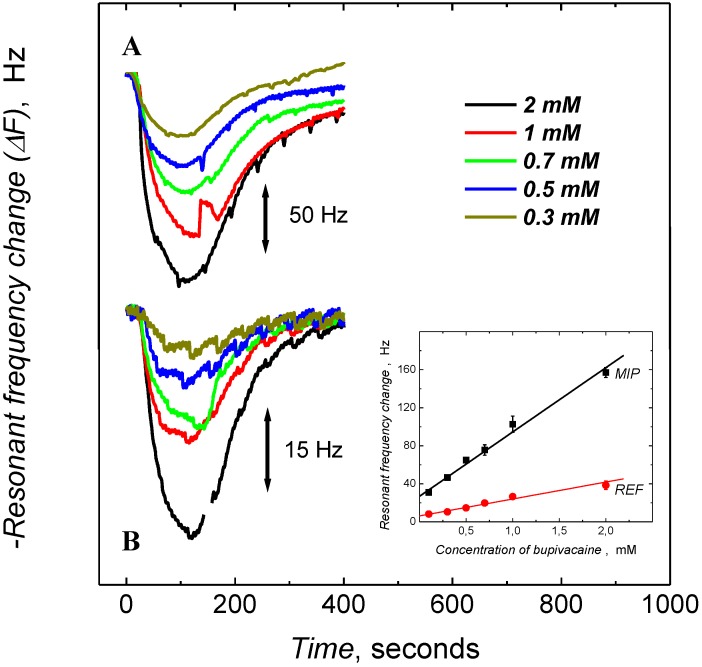
Variation in the resonant frequency of gold-coated quartz resonators overlaid with (**A**) MIP and (**B**) REF films prepared in LC medium, upon injection of analyte (**1**) under FIA condition. Inset is the corresponding FIA calibration plots for **1** on MIP and REF film. Phosphate buffer (0.01 M) containing 150 mM NaCl at pH = 8.5 was used as carrier solution at the flow rate of 25 µL/min. The injection volume was 75 µL.

**Figure A4:**
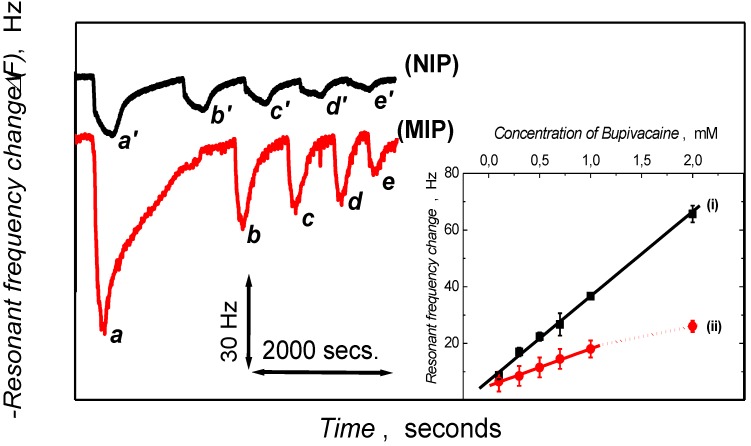
Variation in the resonant frequency of the gold-coated quartz resonator overlaid with bupivacaine-templated MIP and REF (NIP) film, prepared in aqueous conditions, upon injection of analyte (**1**) under FIA condition. Concentration of the analyte injected are (a, a') 2, (b, b') 1, (c, c') 0.7, (d, d') 0.5 and (e, e') 0.3 mM. Inset denotes the FIA calibration plots for (**1**) on MIP (i) and REF (ii) films. Volume of the injected analyte was 50 µL. Phosphate buffer solution (0.01 M) containing 150 mM NaCl at pH = 8.5 was used as carrier solution at the flow rate of 25 µL/min. Volume of the injected analyte was 75 µL.

In Figure 4, the QCM traces (resonant frequency *versus* time) from the injection of **1** over the LC-MIP and REF film coated resonators at concentrations from 0.3 to 2 mM are shown. The response time, the time taken by the signal to reach 90% of its maximum value, was as short as ≈70 s. The sensor surface was recovered by subsequent washing with the running buffer solution until the frequency had been returned to its initial value, 6.5 min. The responses were significantly greater for the MIP surface than the corresponding reference. This was in contrast to the bupivacaine-MIP films prepared in aqueous conditions; which had lower values for both response and recovery times, ≈100 s and 8.3 min, respectively (Figure A4). Collectively these results demonstrate that the hierarchical architectural features present in the MIP film prepared in the presence of the LC medium lead to an enhancement of sensor performance. 

The MIP chemosensor responses were calibrated (Figure 4) for the determination of **1** using the frequency response *vs*. time curves. The dampening of the resonant frequency of the sensor was observed to vary linearly with the concentration of the injected analyte (**1**). Table 1 gives the sensitivity values obtained from the slope of the calibration plots. In the case of the nanostructured MIP film (Inset in Figure 4), sensitivity was over twice that of the non-structured surface prepared, in water (Figure A4) with linearity over the concentration range 0.1 to 2 mM (3–60 μg/mL). In the case of the REF films (both LC and water), the sensor responses were saturated with the analyte (**1**), when the concentration of the analyte exceeded 1 mM (Inset to Figure 4B and Figure A4), and the sensitivities of the reference polymer films were at least half as low as the corresponding MIP film (Table 1). The limit of detection (LOD) of the chemosensor was 100 nM (30 ng/mL) bupivacaine determined under optimized FIA conditions, *i.e*., the sample volume was increased to 1 mL, while the flow rate of the carrier buffer solution was 25 µL/min (signal to noise ratio S/N = 3).

**Table 1:** Relative sensitivities and stability constants, *K*_s_ of the bupivacaine MIP and REF films.

Polymer film (preparation medium)	Relative sensitivity (Hz/mM)^a^	Correlation coefficient	*K*_s_ (M^-1^)^ a^
MIP (H_2_O)	29.92 ± 4.15	0.998	660.56 ± 17.56
REF (H_2_O)	13.22 ± 3.22	0.996	330.12 ± 17.12
MIP (LC)	67.64 ± 4.91	0.995	2187.61 ± 29.02
REF (LC)	17.81 ± 2.67	0.989	444.27 18.71

^a^ Errors represent ± standard deviation.

The binding ability of the MIP film with the analyte was quantified by determining the apparent stability constant (*K*_s_), of the non-covalently bound MIP-analyte (MIP-A_n_) complexes [18,19] (Equation (1)). The affinity interaction between the MIP and the analyte A_n_ is governed by the corresponding association (*k*_a_) and dissociation (*k*_d_) rate constants, and can be derived with the kinetic equation (Equation (2)) as reported elsewhere [48,49,50,51,52,53].

MIP + A_n_ ⇔ MIP − A_n_**(1)

*f* = *f*_eq_ [1 − exp(−*k*_obs_*t*)] (2)

where *k*_obs_ = *k*_a_*c*_T_ + *k*_d_ and *c*_T_ is the concentration of analyte.

**Figure A5:**
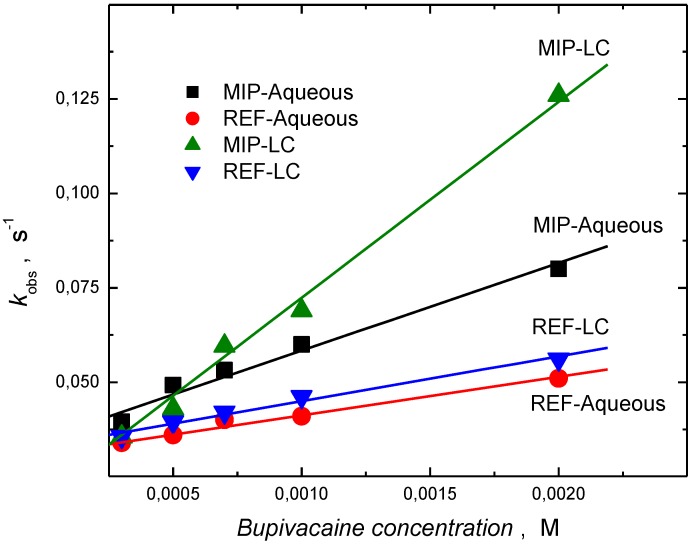
Dependence of observed rate constant, *k*_obs_, on the concentration of (**1**) upon binding with template extracted MIP and REF film prepared in LC medium and aqueous medium, respectively. *k*_obs_ values were calculated by non-linear curve fitting of the binding part of the frequency *vs*. time trace.

By fitting the initial parts of the FIA binding curves (Figure 4) for the different bupivacaine concentrations to Equation (1), the apparent rate constants could be deduced, *k*_obs_ [15,18,19,54,55,56]. The determined *k*_obs_ values varied linearly with the concentration of the analyte (Figure A5). The ratio of the slope (*k*_a_) and intercept (*k*_d_) calculated from this plot gives the value of the apparent stability constant (*K*_s_ = *k*_a_/*k*_d_) [56], for the MIP-analyte complexes (Table 1). Essentially, the stability constant values follow a similar trend to that of the sensitivities, with very high and low values for imprinted and reference polymer, respectively.

**Figure 5:**
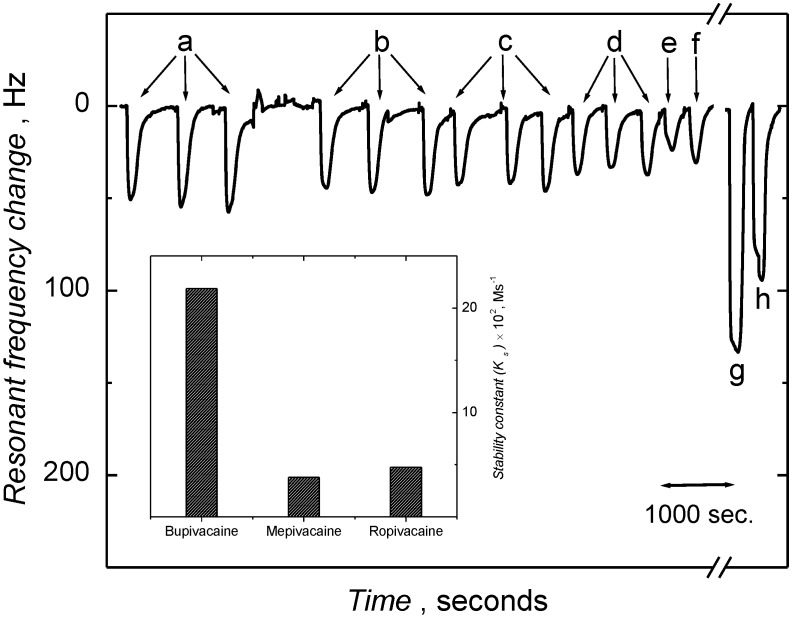
Resonant frequency *versus* time curve for the bupivacaine, and analogues (**5** and **6**) under FIA conditions. Inset is the histogram of stability constant values for MIP-analyte complex. The carrier solution was a phosphoric acid solution (0.01 M) containing 150 mM NaCl at pH = 8.5, with a flow rate of 25 µL/min. An injection volume of 75 µL was used for the analytes mepivacaine ((**a**) 2, (**b**) 1, (**c**) 0.7, (**d**) 0.5, (**e**) 0.1 and (**f**) 0.3 mM) and bupivacaine (1 (**g**) and 1.7 mM (**h**)) concentration.

**Figure A6:**
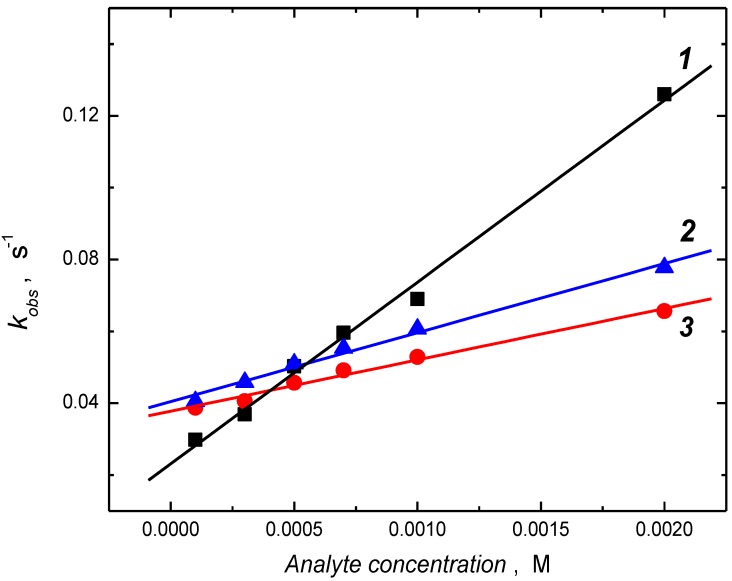
Dependence of observed rate constant, *k*_obs_, on the concentration of analyte (**1**) bupivacaine, (**2**) ropivacaine and (**3**) mepivacaine upon binding with template extracted MIP prepared in LC medium. *k*_obs_ values were calculated by non-linear curve fitting of the binding part of the frequency *vs*. time trace.

To investigate the fidelity of the imprinting induced binding sites, the LC-MIP chemosensor was examined with respect to ligand affinity. This cross reactivity study was performed using the closely related analogs mepivacaine (**5**) and ropivacaine (**6**). These substances differ from **1** by lack of only one (**6**) or three (**5**) methylene residues in the *N*-alkyl side chain. Frequency response *versus* time curves for the repeated injection of these analytes as well as the template (**1**) are shown in Figure 5. The frequency change was noticeably higher for the **1** than for the **5** and **6**, highlighting the greater affinity for bupivacaine. The relative sensitivity values (Table 2) reflecting the affinities, were three and two times higher for **1** than that for the interferants (**5** and **6**), respectively. The stability constant values (determined from the apparent rate constant *k*_obs _values, Figure A6) for the MIP-analyte complexes reveal the high affinity and selectivity (Inset to Figure 5) of the MIP chemosensor for bupivacaine (**1**). It is important to note that the ligand affinity for the and detection limits of the present MIP-QCM sensor assembly presented here are comparable to that of HPLC methods [57,58,59,60] and superior to those of electroanalytical (ion selective electrodes) methods [61] reported for the selective determination of bupivacaine (Table 3). 

**Table 2:** Relative sensitivities and interference factors of the bupivacaine LC-MIP films.

Analyte	Relative sensitivity Hz/mM	Correlation coefficient	Interference factor, IF ^a^
Bupivacaine (**1**)	67.6 ± 4.91	0.995	1.00
Mepivacaine (**5**)	28.4 ± 1.51	0.989	0.17
Ropivacaine (**6**)	38.0 ± 5.16	0.990	0.22

^a^ Interference factors (IF) were calculated by normalizing the stability constant values to that of MIP-bupivacaine complex.

In summary, the bupivacaine selective chemosensor based upon a MIP recognition film prepared in the LC medium exhibits good selectivity and sensitivity. The hexagonal cylindrical structures present in the LC medium [36] affords imprinted polymers with nanowire or fiber-like structures [62,63]. LC media have even been claimed to enhance the polymerization process due to improved solubility of monomers [64,65] and radicals [66]. Furthermore, the polymer films synthesized in the LC medium demonstrated high stability. 

**Table 3:** Comparison of analytical methods for the determination of bupivacaine.

Method	Reported dynamic concentration range	Detection limit, LOD	Reference
Present work	3–60 μg/mL	30 ng/mL	-
HPLC	5.0–50 μg/mL	250 ng/mL	[58]
HPLC	0.02–5.00 mg/L	20 μg/L	[60]
HPLC	0.033–3.31 μg/mL	10 ng/mL	[67]
Ion-selective electrodes	16 μM–100 mM	-	[61]

## 4. Conclusions

Nanostructured bupivacaine-selective molecularly imprinted 3-aminophenylboronic acid-*p*-phenylenediamine co-polymer films have been prepared on gold-coated quartz (Au/quartz) resonators by electrochemical synthesis under cyclic voltammetric conditions in a liquid crystalline (LC) medium (Triton X-100). Films prepared in water and in the absence of template were used for control studies. The presence of the LC-derived nanowire or brush-like structures on the sensor surface enhanced the performance of the piezoelectric microgravimetric (QCM) sensors. Importantly, the performance of this sensor system compared well with those of other analytical techniques. Finally, the facile fabrication together with the significant enhancement in sensor sensitivity highlight the potential of this soft sacrificial LC-based imprinting strategy for the fabrication of polymeric materials with hierarchical architectures, which are of particular interest for use in surface-dependent application areas, e.g., biomaterials or sensing [67].

## Appendix

**Figure A1:** Cyclic voltammograms.

**Figure A2:** UV-vis spectra from template elution studies.

**Figure A3:** SEM studies.

**Figure A4:** QCM studies of MIP and REF.

**Figure A5:** Concentration dependence of rate constants (**1**).

**Figure A6:** Concentration dependence of rate constants (**1**, **5** and **6**).
